# The physical sequelae of perinatally acquired HIV in adolescents: a research proposal

**DOI:** 10.1186/s13104-019-4079-5

**Published:** 2019-01-28

**Authors:** Nicolette Comley-White, Joanne Potterton, Veronica Ntsiea

**Affiliations:** Department of Physiotherapy, School of Therapeutic Sciences, Faculty of Health Sciences, University of the Witwatersrand, 7 York Road, Johannesburg, 2193 South Africa

**Keywords:** HIV, Perinatally infected, Adolescents, Physical

## Abstract

**Objectives:**

As the global access of antiretrovirals for HIV-infected infants improves, so the body of perinatally HIV-infected adolescents (PHIVA) grows. The neurological and physical complications of HIV, both in children and in adults, are well established, however there is a paucity of data pertaining to PHIVA, a group of people who have had a lifetime exposure to the virus and to antiretrovirals. There has been a resounding call for further research in this area, as well as for the development of policies and programmes for this population. The aim of this study is to determine the physical sequelae in PHIVA and to propose a model of care for this population.

**Methods:**

Through interviews with PHIVA, the perceived physical challenges will be established. Thereafter a cohort study with age-matched participants will determine if PHIVA have any limitations in fatigue, endurance, motor function and muscle strength, body mass index, peripheral neuropathy, level of disability and quality of life. Using these results, a model of care will be proposed through the nominal group technique with both PHIVA and clinicians working in HIV and adolescence.

## Introduction

Current data shows that AIDS-related deaths have declined by 48% from 2005 to 2016 [1], however the human immunodeficiency virus (HIV) remains the second leading cause of death in adolescence globally [2] and the leading cause in Africa [3]. The global scale-up of antiretroviral therapy (ART) in the management of HIV has shifted the paediatric HIV pandemic to chronic disease management, with a growing body of perinatally HIV-infected adolescents (PHIVA) [4]. In 2016 there were an estimated 2.1 million adolescents aged 10–19 years living with HIV, with 84% living in sub-Saharan Africa [3] and although perinatal HIV transmission forms the majority of infection route, the specific number of PHIVA is unknown [4].

While the paediatric brain has strong neural plasticity, it also has a degree of neural vulnerability where adverse environmental factors can impact on normal development [5, 6]. One of these adverse factors can be the HI virus, which is able to cross the blood brain barrier and infiltrate the central nervous system causing neurocognitive impairment [7]. Even with the early use of ART many children with HIV still present with neurodevelopmental problems due to neuroinflammation, vascular dysfunction and hypercoagulability [8]. In addition to damage done to the developing nervous system by the HI virus, the long term use of ART needs to be considered. Hepatotoxicity, mitochondrial toxicity, skin toxicity, hypersensitivity and lipodystrophy are some well-known adverse effects of ART [9, 10]. Depending on the class of drug and the individual, a variety of neurotoxic adverse effects exist from ART, including mania, psychosis, insomnia, irritability, vivid dreams [11].

Some of the established challenges that children and adults with HIV face are peripheral neuropathy, fatigue, decreased endurance and muscle strength, motor function impairments and body mass index changes [12–21]. Concerning these challenges, there is little data available on what PHIVA face.

With the advent of ART and its increased availability to perinatally infected HIV positive children, there is a growing number of adolescents living with HIV and ART use since birth, facing a lifetime of chronic illness management [22–25], the consequences of which are still being established [4]. Overall, there is a lack of data available for the challenges that PHIVA face [26]. Of the studies that have been done on neurodevelopment in PHIVA, the majority are from resource-rich countries, and not from resource-scarce countries (where PHIVA are more likely to face running a child-headed household; have poorer access to health care; face poverty, deprivation and opportunistic infections [26]), where there is the highest prevalence of PHIVA [26].

The literature has identified a clear need for further research to be done in the area of neurodevelopment in PHIVA (especially in resource-scarce countries), as well as for the urgent development of policies and programmes to cater for PHIVA [26–28]. Although models of care for different areas of managing HIV are available [29–32] to date there is no literature presented on suggested models of care for PHIVA. Furthermore, since the challenges that PHIVA face is a relatively new area of concern, any existing models of care may well not be sufficiently responsive to this population, as is often the case with existing models of care based on historical information [33].

The aim of this study is, over three phases, to determine what the perceived challenges are that PHIVA face due to physical sequelae of HIV; to then establish these challenges clinically; and lastly to propose a model of care for PHIVA. This manuscript will provide the outline of the research proposal used to meet the study’s intentions.

## Main text

The study is designed as a mixed methods study over three phases. In the first phase qualitative information will be gathered through semi-structured individual interviews. The second phase is designed as a cohort study with a comparison group of age-matched participants. In the third phase, a model of care for PHIVA will be proposed and consensus will be obtained through the nominal group technique.

### Subject selection

Adolescence is defined as ages 10–19 years [2, 34], with early (10–15 years), middle (14–17 years) and late (16–19 years) stages [35]. For the purposes of this study, early and middle phases of 10–14 years and 15–17 years respectively will be used (late phase adolescents will not be included due to the age limitations of the outcome measures). Participants will be recruited from an existing cohort of PHIVA involved in a clinical trial looking at the chronic effects of growing up with HIV: the childhood HAART alterations in normal growth, genes and aGing evaluation study (CHANGES) (M120871). To date CHANGES has approximately 255 PHIVA (aged 10 and above) attending a clinic every 6 months, as well as a data base of HIV negative adolescents, providing the ideal source of participants for this study. The CHANGES trial is based at Rahima Moosa Mother and Child Hospital (RMMCH), Johannesburg, South Africa. Data routinely collected for CHANGES includes (but is not limited to) the participant’s body mass index, quality of life scores, clinical data (such as viral load, CD4 count etcetera).

This study has been approved by the Human Research Ethics Committee of the University of the Witwatersrand (certificate number M180226), and registered with the South African National Health Research Database (reference GP_201806_010). Written, informed consent, assent and permission will be obtained from the necessary parties.

### Sample size and inclusion/exclusion criteria

For the first phase, data will be collected for participants in two groups, namely early adolescence and middle adolescence. This will continue until data saturation is reached in each age group. Initially eight participants per age group (i.e. 16 participants) will be invited to participate and booked for interviews. Adolescents aged 10–17 years will be invited to take part in the study, but will be excluded if they have physical and/or cognitive impairments rendering them unable to participate in the interview process or if they have impairments not related to HIV (e.g. traumatic brain injury).

For phase two, the inclusion and exclusion criteria remain the same, but in addition, age-matched HIV negative participants will be invited to participate as the age-matched, comparison cohort. To date, CHANGES has an age appropriate population of 255 HIV positive participants. These adolescents are representative of the community of PHIVA. A sample of 154 HIV positive participants would give a confidence level of 95% (5% margin of error). A further 154 HIV negative participants would comprise the age-matched, comparison cohort, thus a total of 308 adolescents would be required for phase two of this study. (Sample size was calculated using Raosoft, Inc^©^ [36]).

Based on the findings of phase one and two, phase three will involve the development of a model of care for PHIVA. This will be put forward for consensus using the nominal group technique (NGT) to two populations, namely a group of PHIVA (sourced from CHANGES), and a group of clinical experts in the field of HIV and adolescents. During phase one and two the principal investigator (PI) will take note of the clinical experts encountered at RMMCH. Snow-ball sampling will also be used to source participants as the clinical experts, who would need to have worked with adolescents with HIV as a nurse, medical doctor, physiotherapist or occupational therapist. It is recommended that five to nine people are used per group for NGT [37].

### Study procedures

#### Phase one

Adolescents and their parent/guardian will be approached and invited to participate in phase one on the day that they are attending the clinic. Through semi-structured interviews participants will meet with the PI individually to explore what the participant perceives as challenges due to the physical sequelae of HIV and how this impacts on their activity and participation levels. The interviewer will use open-ended questions to explore what the participant enjoys doing socially; what they find physically difficult; what they feel that they are not able to do that their friends can do; what their attitude is towards their health etcetera.

The interviews will be audio-recorded and transcribed verbatim at a later stage. The PI will take field notes during the interview and at the end of each interview the PI will capture the essence of the interview session [38].

#### Phase two

Participants and their guardians will be invited to take part in phase two of the study on the day that they attend the clinic. After they complete their doctor’s appointment, they will meet with the researcher and data will be collected from the participants as per Table 1.

**Table 1:** Data collection parameters for phase two

Data collected during clinical assessments	Outcome measure tool	Explanation of outcome measure
Fatigue	HIV related fatigue scale	A 56-item questionnaire used to establish levels of fatigue in people with HIV
Endurance	Six minute walk test	A measure of distance walked in 6 min to establish a person’s endurance
Motor function impairment	Movement assessment battery for children—second edition (movement ABC-2)	A tool used to assess fine and gross motor impairment in children and adolescents
Peripheral neuropathy	Brief peripheral neuropathy screen	A tool used to assess peripheral neuropathy in people with HIV
Muscle strength	Standing broad jump	An index of muscular fitness in children and adolescents established through three attempts at a standing broad jump
Disability	World Health Organisation disability assessment schedule for children (WHODAS-Child)	A 36-item questionnaire for assessing disability in children and adolescents through rating levels of difficulty in daily activities

Data collected from patient files	Outcome measure tool	Explanation of outcome measure
Quality of life (QOL)	Pediatric quality of life inventory version 4.0 (PedsQL™ 4.0)	A 23-item tool establishing the quality of life in children and adolescents with chronic illness
Body mass index	Weight/height squared	A measurement of weight in relation to height
Clinical data	CD4; nadir CD4; viral load; age at initiation; drug regime; key co-morbidities	Clinical data pertaining to the participant’s HIV management

#### Phase three

Data gathered in phase one and two will inform phase three: the development of a model of care for PHIVA. The model of care will be put forward for consensus via the NGT. Two groups of five to nine participants [37] will be used: one of PHIVA and one of clinical experts (as described in the inclusion criteria). The participants and PI will meet on an allocated date and the NGT structure will follow the method as described in the literature, namely, to give an explanation of the purpose and procedure; allow for the silent generation of ideas; participants then share ideas; a group discussion; followed by voting and ranking to prioritise the ideas [37].

**Fig. 1:**
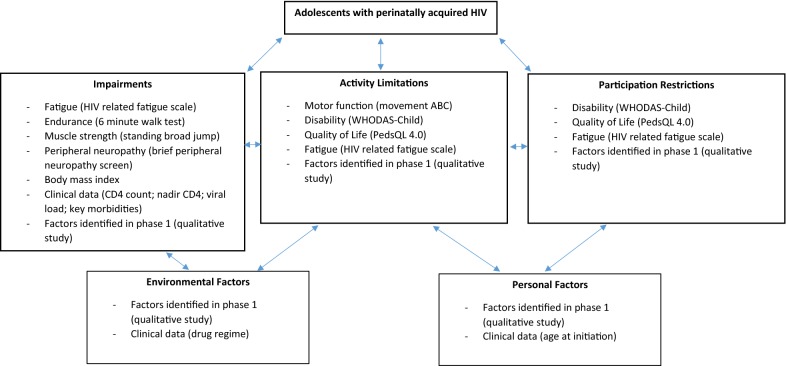
Conceptual framework for the physical sequelae of perinatally acquired HIV in adolescents [39]

The application of the international classification of functioning, disability and health (ICF) as a conceptual framework for disability has been described in the literature and is used as a common language in discussing disability [39]. Based on this, this study will utilise the ICF as its conceptual framework (as shown in Fig. 1). The impairments, activity limitations and participation restrictions identified in phase one and two will feed into the development of the model of care for phase three.

### Data analysis

The data from the interviews will be organised, coded and analysed by the PI and another coder. Substantive statements will be identified in the transcribed interviews and thematic analysis will be undertaken using the general inductive approach to analyse the data [40], with manifest analysis being used to quantify the content [41]. Thereafter the coders will discuss the material and identify the themes, categories and sub-categories [41]. This improves the validity through triangulation [41]. The field notes taken during the data collection will be an added aspect of triangulation.

Data collected in phase two will be transferred to Microsoft Excel™ 2016 where frequencies, means and standard deviations will be calculated for descriptive statistical analysis. Statistica™ will be used to analyse the data further. The authors hypothesise that the age-matched, comparison cohort will acquire better scores. P values of < 0.05 will be considered statistically significant. T tests will compare clinical data, demographic data and data from the outcome measures. Mean composite scores will be compared between the PHIVA and age-matched, comparison cohort. To measure the variable difference between the two groups the Chi squared test and Kruskal–Wallis one-way ANOVA will be used. A logistic regression will be used to analyse factors associated with disability.

For phase three, due to the nature of the ranking, NGT yields both quantitative and qualitative data [42]. The qualitative data will be analysed inductively (as per phase one) and the ranking of items will be analysed as quantitative data using descriptive statistics with frequencies and means.

Table 2 gives a summary of the methodology for the study across the three phases.

**Table 2:** Summary of the methodology for the study across three phases

	Phase one	Phase two	Phase three
Study design	Semi-structured interviews	A cohort study with a comparison group of age-matched participants	Consensus for a model of care using NGT
Source of participants	CHANGES at RMMCH (for phase three medical professionals will be sourced from the site)		
Sample size	Eight participants per group; 2 groups (early and middle phase adolescence: ages 10–14 and 15–17 years, respectively)	308 (154 HIV+ and 154 HIV− [matched]) participants	Five to nine participants per group; 2 groups (representatives of PHIVA and of clinical experts)
Inclusion criteria	PHIVA in CHANGES, aged 10–17 years	Adolescents aged 10–17 years (HIV+ and −)	PHIVA in CHANGES, aged 10–17 years Interdisciplinary team member in field of HIV and adolescence
Exclusion criteria	Physical/cognitive impairments preventing their participation Physical/cognitive impairment not due to HIV		
Procedure and instrumentation	Individual, semi-structured interviews	HIV related fatigue scale Six minute walk test MABC-2 Brief peripheral neuropathy screen WHODAS-child Standing broad jump PedsQL 4.0 Body mass index	Nominal group technique for consensus on a model of care [37]
Data analysis	Identify substantive statements Inductive approach Manifest analysis Separate coding done to identify themes, subthemes and categories	Descriptive statistics Appropriate tests of association Logistic regression analysis	Qualitative data: inductive analysis as per phase one Quantitative data: descriptive statistics of averages and means

### Conclusion

Establishing the perceived and actual physical sequelae of perinatally transmitted HIV in adolescents will address one of the gaps in knowledge that we have as clinicians working with PHIVA. Creating a model of care will assist health care providers in early identification, assessment and management of potential physical problems, including the necessary referral of the PHIVA to relevant members of the interdisciplinary team. By addressing the challenges, one hopes to improve the quality of life and community participation of PHIVA. Informing this research with the voice of the affected population in the assessment and development of the model of care ensures that we are working for adolescents, with adolescents, and thus potentially optimising the uptake of the model of care.

## Limitations

Although the study site has a large feeder area, taking participants from only one site results in a somewhat homogeneous population and thus may not produce results generalizable to the global population. However, this study will provide the starting point for determining the physical sequelae in PHIVA and the basis of a model of care for this population, which could be tested in further research.

Furthermore, the HIV related fatigue scale has not yet been used in adolescents, nor tested across all cultures, however, it remains one of the few tools that are available for the use of establishing fatigue in HIV [43].

## Abbreviations

ART: antiretroviral therapy
CHANGES: childhood HAART alterations in normal growth, genes and aGing evaluation study
HIV: human immunodeficiency virus
ICF: international classification of functioning, disability and health
Movement ABC-2: movement assessment battery for children—second edition
NGT: nominal group technique
PedsQL™ 4.0: pediatric quality of life inventory version 4.0
PHIVA: perinatally HIV-infected adolescents
PI: principal investigator
QOL: quality of life
RMMCH: Rahima Moosa Mother and Child Hospital
WHODAS-Child: World Health Organisation disability assessment schedule for children
